# The electrical characteristics of Nanosheet FET within the quasi-ballistic transport: Role of scattering and temperature variation

**DOI:** 10.1371/journal.pone.0350021

**Published:** 2026-05-26

**Authors:** Shubham, Rajan Kumar Pandey

**Affiliations:** Department of Micro and Nano Electronics, Vellore Institute of Technology, Vellore, Tamil Nadu, India; Gazi University, TÜRKIYE

## Abstract

The article highlights the effects of quasi-ballistic and diffusive transport on electron mobility, band gap, and electrostatic behavior in scaled Nanosheet FETs. It also provides comprehensive physical insight into the effects of different scattering mechanisms on the gate-length scaling process (from 16 nm to 6 nm) and their impact on performance metrics. The temperature is modeled from 220 K to 450 K to analyze its effect on electron mobility and the lateral electric field profile. It is observed that defects, such as oxygen vacancies, affect the work function of the gate stack region and induce scattering mechanisms at the oxide interface, thereby enhancing band-to-band and trap-assisted tunneling of electrons. Increased temperature in the device causes significant phonon scattering, resulting in approximately a 45% drop in the mobility, with a standard deviation of 328.7 cm²/V·s. The higher phonon and surface-scattering rates at elevated temperatures modify the band gap profiles, leading to a reduction of 48–72 meV in the band gap. Due to the reduced scattering and lower contact-poly pitch, the highest drive current is achieved at a 6 nm gate length in the quasi-ballistic and diffusive transport regimes. Gate-length scaling suffers from increased scattering rates and higher tunneling probabilities, leading to higher leakage current and a reduced I_ON_/I_OFF_ ratio. Scattering mechanisms introduce resistance in the channel region, resulting in a drop in mobility. The electrostatic profiles and mobilities are mapped along the channel to comprehend the device operation at the scaled node.

## 1. Introduction

In the constant pursuit of high speed, low power consumption, and minimized chip area, traditional MOSFET-based logic devices have shifted from FinFETs to stacked nanosheet field-effect transistors (NSFETs). NSFET-based logic devices demonstrate superior electrostatic control and enhanced drive current within the same device footprint compared to existing FinFET-based logic devices [1–3]. The transition from FinFET to gate-all-around Nanosheet FET (NSFET) represents a significant advancement in the device technology roadmap to sustain Moore’s law at sub-3 nm technology nodes, as projected by the International Roadmap for Devices and Systems (IRDS) [4]. NSFETs are expected to replace FinFET-based logic devices at sub-3 nm nodes due to their enhanced scalability and superior electrostatic control over the channel region [5,6]. It offers improved power-performance-area (PPA) metrics, reduced short-channel effects, and enhanced drive current. The gate-all-around structure, where the gate wraps the channel on all four sides, significantly reduces leakage current, a critical factor for low-power operation. In contrast, FinFET devices at scaled nodes suffer from increased process-induced variability, resurfacing of short-channel effects, and self-heating [7,8]. Additionally, improving drive current in FinFETs is challenging due to fin quantization, where fin width is constrained by fin height and thickness, limiting layout flexibility and causing threshold voltage (V_T_) variations that degrade reliability. NSFET overcomes fin quantization limitations by enabling multi-channel stacking in both vertical and horizontal directions, thereby increasing the effective device width and improving drain current. It provides flexibility in channel-width engineering, allowing the use of wider nanosheets for high-performance (HP) applications and narrower nanosheets for high-density (HD) applications. This tunability enables trade-offs among key performance metrics such as drive current, capacitance, and device footprint, while reduced channel width also lowers parasitic capacitance. Furthermore, NSFET employs a full-bottom dielectric isolation scheme to suppress leakage paths and eliminate sub-fin leakage current [9,10]. NSFET enables the standard-cell scaling to 5-track (5T and below), enabling further density scaling. However, despite these advantages, IRDS projections indicate that improvements in energy per switching will remain limited, while power density, variability, and interconnect resistance emerge as dominant bottlenecks. Process variation issues, such as metal gate granularity (MGG) and line-edge roughness (LER), are expected to dominate random dopant fluctuation (RDF) in undoped channels [11]. The reduced physical geometry in the device creates a phonon-confinement region and enhances phonon-boundary scattering. During scattering, electrons interact with acoustic and optical phonons, releasing energy into the crystal lattice and increasing the electron temperature (eTemp), which is more than the ambient temperature [12–14]. Also, integrating low-thermal-conductivity materials and a silicon-on-insulator design into the process technology traps the generated heat within the device, hindering heat dissipation and causing localized thermal hotspots that eventually degrade performance and reliability [15,16]. Although NSFET shows strong performance potential, it also introduces significant manufacturing challenges, including advanced etching processes and the formation of inner spacers, which increase fabrication complexity and cost [17–19]. The GAA nanosheet FETs are expected to sustain CMOS scaling for approximately a decade, after which the logic device roadmap transitions toward more advanced architectures such as Complementary FET (CFET), Forksheet FET, and 3D integration schemes [20–24].

The stacked NSFET-based logic devices at the 5/3 nm node face performance degradation due to quantum confinement (QC) effects and diffused resistance in the source/drain epitaxial regions. Downscaling the channel region in the stacked NSFET results in significant geometric confinement, thereby increasing the effective mass and bandgap [25]. This causes splitting in the energy bands and the formation of multiple sub-bands. The QC effects also reduce the availability of electron and hole energy states in the channel material (density of states) and decrease the inversion charge-carrier density, which impacts the I_D_ and, overall, degrades the electrostatic characteristics of the device [25,26]. The carrier mobility improves in the ballistic transport model due to reduced intervalley phonon scattering in nanoscale transistors. However, surface effects and electron-electron interactions in confined dimensions may degrade mobility due to surface roughness and Coulomb scattering. Electronic transport in the stacked NSFET can occur through one-dimensional subbands, utilizing advanced transport mechanisms such as the NEGF or the semiclassical sub-band Boltzmann transport equation (sub-BTE). The NEGF transport mechanism accounts for the wave nature of electrons, requiring detailed information about the propagation of the electron wave packet in nanoscale devices, thereby increasing computational costs [27]. The self-energy terms are primarily non-local functions of the spatial coordinate in the NEGF transport formalism. Consequently, it is challenging to compute scattering mechanisms other than electron-phonon interactions [28]. Therefore, the sub-BTE, in conjunction with diffusive transport, is preferred for predicting reliable electrical characteristics in nanoscale devices. The sub-BTE approach serves as an intermediate solution between complete quantum transport and semiclassical BTE transport. It considers the confinement effect in the non-local and cross-sectional regions and semi-classically describes the transport phenomenon in the sub-bands [28,29]. The formulation of the sub-BTE transport mechanism is based on the self-consistent solution of the two-dimensional Schrödinger equation, coupled with the Poisson equation. In essence, the sub-BTE transport mechanisms throw light on electronic wave functions, subband energy levels, and self-consistent potential profiles. However, on the downside, ionized scattering and subband dielectric screening increase computational cost.

Despite the high computational cost, it is essential to study the device's electrical characteristics using a sub-BTE alongside the traditional diffusive transport mechanism to understand the trade-offs between device design and performance. This work formulates a simulation model that combines the sub-BTE and diffusive transport mechanisms. We investigated the impact of ambient temperature variations, gate-length scaling, and atomistic defects (oxygen vacancies in high-k oxides) on the electrical performance of NSFET within the sub-BTE and diffusive transport models. The terminal parameters, such as threshold voltage (V_T_), drain current (I_D_), sub-threshold swing (SS), and off-state leakage current (I_OFF_), are computed to elaborate and compare the device performance. The physical significance of remote Coulomb, surface roughness, and phonon scattering on performance metrics, namely electron mobility (eMob), electric field, and bandgap, is discussed. Section 2 discusses the design framework, design steps, and the simulation models. Section 3 and its subsequent subsections provide insights into the simulation results, followed by a conclusion in Section 4.

## 2. Design & physical models

**Fig 1:**
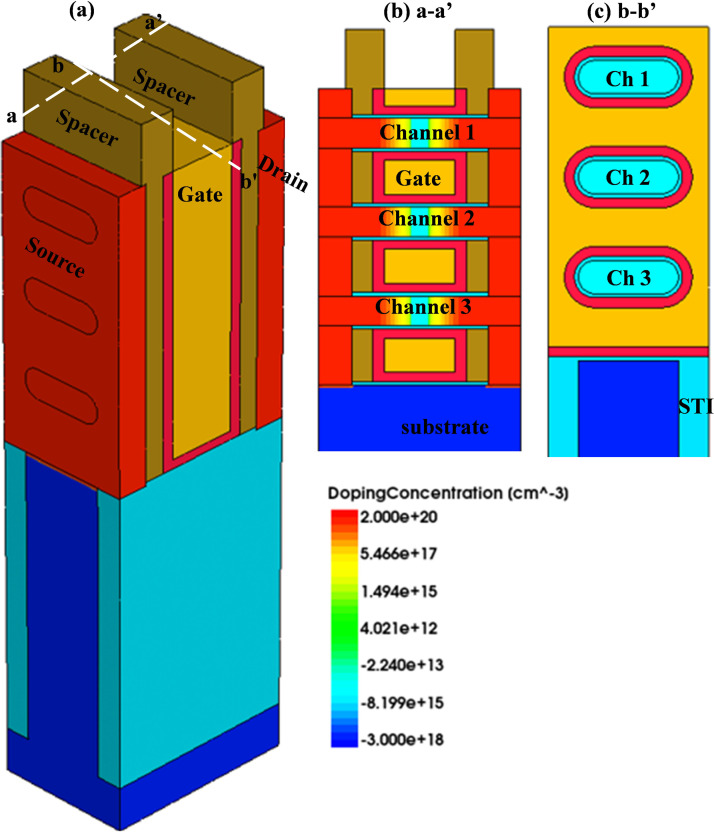
Designed profiles of the (a) bulk 3D stacked Nanosheet FET, (b) 2D- cross-sectional A-A’ profiles along the channel, and (c) 2D- cross-sectional B-B’ profiles across the channel.

**Fig 2:**
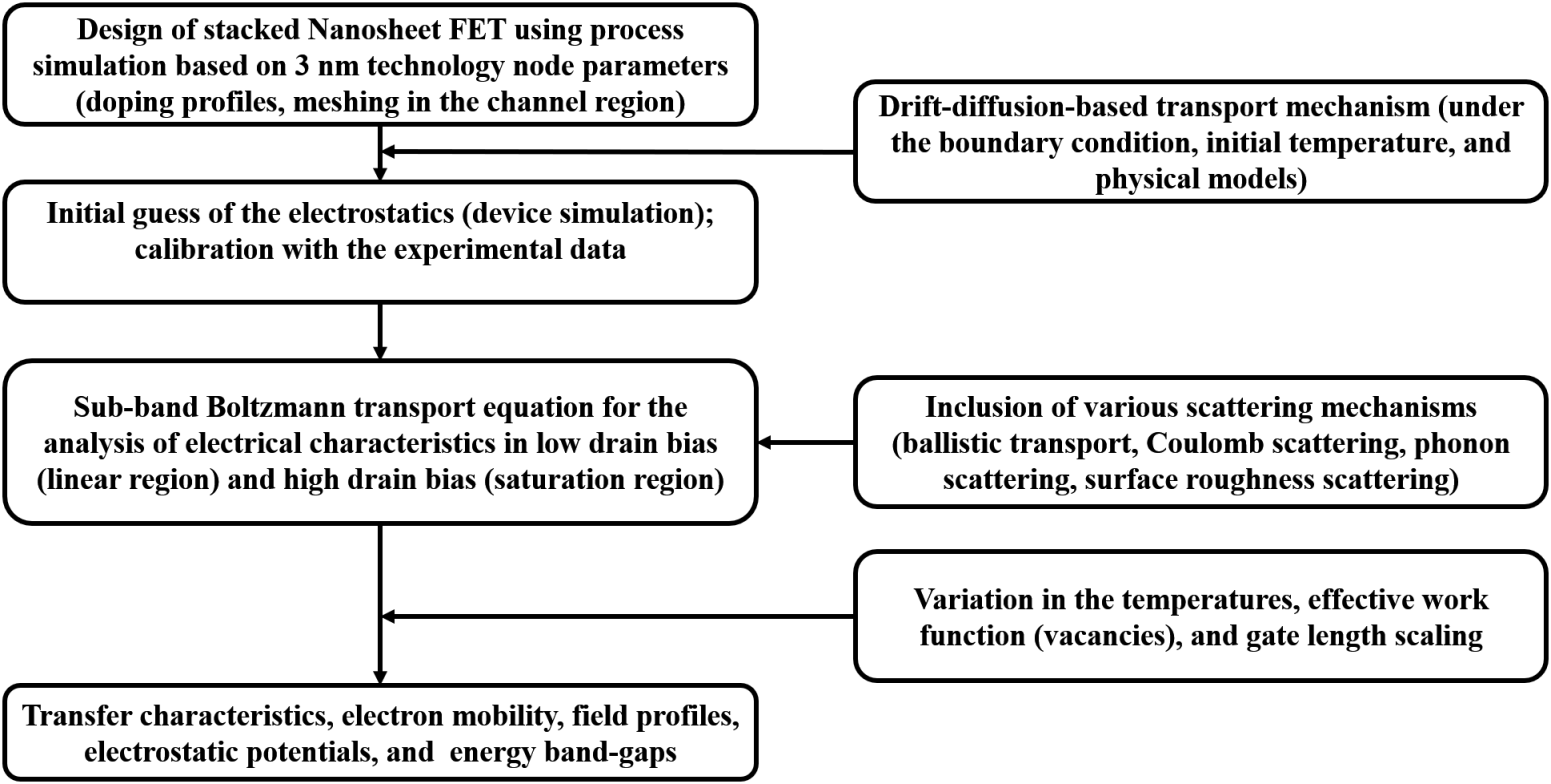
Device design and simulation modeling setup using process and TCAD framework [30,34].

The baseline NSFET device is designed as per the 3 nm technology node suggested in IRDS, and adapted from [30]. Calibrated 3D TCAD models are used to study the transport mechanisms and electrical behavior of the NSFET-based logic device. The designed baseline device and its cross-sectional profiles along and across the axes are illustrated in Fig 1, and a detailed design parameters are summarized in Table 1. The stacked NSFET is designed using the process fabrication steps proposed in [31,32]. While designing the NSFET device, the channel is assumed to have a < 110 > crystal orientation, and the top surface orientation of the deposited nanofilm slabs is < 100 > . The gate length and the sheet width of the NSFET are 12 nm and 15 nm, respectively, while the sheet thickness is 5 nm. An interfacial silicon oxide layer of 6 Å (0.6 nm) thickness is deposited on the silicon layer, and a 15 Å (1.5 nm) thick HfO_2_ is deposited anisotropically as the high-κ oxide. Titanium nitride (TiN) is deposited as the gate metal and an effective work function of 4.62 eV for the gate stack region is chosen in this study for the defect-free states (oxygen vacancies and interstitials) based on our previous work using the ab-initio calculations [33]. The design setup and simulation framework are presented in Fig 2.

**Table 1:** Designed parameters for the baseline stacked NSFET device.

Parameters	Values
Gate length	12 nm
Sheet height	5 nm
Interfacial oxide thickness	6 Å (0.6 nm)
High-κ oxide thickness	15 Å (1.5 nm)
Channel orientation	<110>
work function (WF)	4.62 eV
drain & source doping concentrations	2 × 10^20^ cm^-3^
channel doping concentrations	1 × 10^15^ cm^-3^

In this study, the two-band k.p model (ellipsoidal) model for electrons and the six-band k.p model for holes are considered to cater to the sub-BTE transport mechanism. The OldSlotBoom model from Sdevice invokes the band-gap narrowing effects, which directly impact the intrinsic carrier density in the conduction region [35,34]. The Philips unified mobility model (PhuMOB) is included in studies of diffusive transport because it combines the minority- and majority-carrier concentrations in the device. PhuMOB considers electron-hole scattering and the temperature dependence of charge-carrier mobility. It also screens the ionized impurities of the charge carriers and accounts for the congregation of the impurities in the transport mechanisms. The Enormal model is utilized to account for the degradation of carrier mobility at the semiconductor-insulator interface. The sub-BTE accounts for the strong quantum confinement (QC) region and quasi-ballistic transport in the channel region of nanoscale devices (nanosheets and nanowires). The scattering mechanisms, including models such as phonon, Coulomb, and surface roughness, are examined to investigate their impact on the transport mechanisms. One intervalley acoustic phonon scattering in electrons, one intervalley optical phonon scattering, and three f- and three g-type intervalley inelastic process-based scattering models are included for the phonon scattering in electrons. In contrast, the optical and acoustic phonon scattering is comprehended for the holes. The exponential model from the power spectrum density function is included for both holes and electrons to account for surface roughness scattering. The contact resistance is lumped into a 3.5 × 10^−9^ Ω/cm^2^ resistance via a Neumann boundary condition at the source and drain electrodes [16].

The solution of the Schrödinger equation on the two-dimensional slices of the channel provides the subbands, which the Sub-BTE solves, and predicts the distribution of charge carriers in each subband. It also solves the Schrödinger equation to obtain the wave functions and predicts the charge-carrier density. When the QC is in two dimensions, the Sub-BTE is concentrated in the channel direction of each subband. The solution of the Sub-BTE considers the *z-direction* as the channel axis and *n* as the number of subbands; the Sub-BTE can be expressed as Eq.1 The Sub-BTE solution predicts the channel coordinates and distribution factor as a function of the k Vector for each subband.


$$\begin{array}{c} -\frac{\partial f_{n}}{\partial k}\frac{\text{1}}{\hbar }\frac{\partial E_{n}}{\partial z}+\frac{\partial f_{n}}{\partial z}\frac{\text{1}}{\hbar }\frac{\partial E_{n}}{\partial k}=S_{n,\text{ in }}-S_{n,\text{ out }} \end{array}$$ (1)

In this equation, $E_{n}$ is the dispersion for the subbands and $f_{n}$ is the distribution function [27]. From the Fermi-Dirac statistics, the in-and-out scattering term ($S_{n,\text{ in }}$– in the scattering term and $S_{n,\text{ out }}$– out scattering term) are given in Eq. 2 and Eq. 3.


$$\begin{array}{c} S_{n,\text{ in }}=\sum_{n^{\prime }}\;\frac{\text{1}}{\text{2}\pi }\int \;dk^{\prime }S_{n^{\prime }n}\left(k^{\prime },k\right)f_{n^{\prime }}\left(z,k^{\prime }\right)\left[\text{1}-f_{n}(z,k)\right] \end{array}$$ (2)

$$\begin{array}{c} S_{n,\text{ out }}=\sum_{n^{\prime }}\;\frac{\text{1}}{\text{2}\pi }\int \;dk^{\prime }S_{nn^{\prime }}\left(k,k^{\prime }\right)f_{n}(z,k)\left[\text{1}-\overset{\prime }{\underset{n}{f}}\left(z,k^{\prime }\right)\right] \end{array}$$ (3)

In these equations, $S_{nn^{\prime }}\left(k,k^{\prime }\right)$ is the total transition time due to the scattering. It is observed that most of the scattering occurs at the channel/drain and source/channel interfaces; therefore, the sub-BTE is solved in the Source/Drain extension and at the interfaces to mitigate the eMob degradation in these regions. The sub-BTE model is solved using coupled Poisson and diffusive transport solutions to predict the total electric current, electrostatic profile, current spectrum, and charge-carrier velocity in the device. For the extraction of device parameters in the saturation region, the drain and gate voltages (V_D_, V_G_) are set to 0.7 V. The drain supply is 0.05 V for calibration in the linear region. Vt is extracted using a constant current extraction mechanism [36], and it is extracted at the biasing at which I_D_ = ($\frac{Weff}{Lg})\times \;Io\;(nA),\;Io=\text{1}\text{0}\text{0}\;nA$ [11,37]. While the drain current (I_D_) is the on-state current at V_DS_ = V_GS_ = V_DD_, and the off-state current (drain leakage) in the device is computed at V_DS_ = V_DD_ and V_GS_ = 0 V In our study, we have set the gate supply (V_GS_) and drain voltage (V_DD_) to 0.7 V each.

## 3. Results

**Fig 3:**
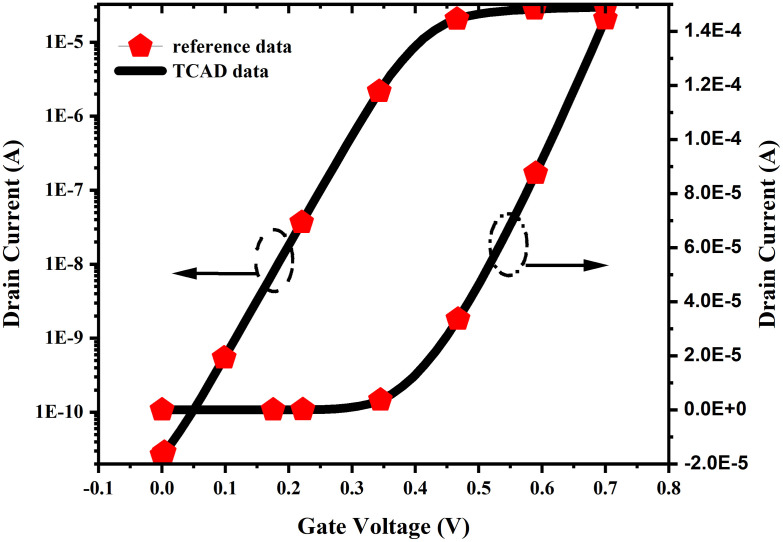
Calibration of transfer characteristics (I_D_-V_G_) of the designed NSFET with the experimental device at high drain and gate bias conditions. The left y-axis shows the current for the linear region, and the right y-axis shows the current for the saturation region.

The transfer characteristics (Id-Vg) of the designed device in TCAD are calibrated against the fabricated stacked NSFET device, and a close match in the calibration characteristics is shown in the Fig 3, which ensures the reliability and accuracy of the simulated results. The physical dimensions of the device are scaled down after the calibration to cater to the 3 nm technology node. The effective work function and tunneling mass of the charge carriers are tuned within the experimental range to match the experimental calibration [1].

### 3.1. Impact of various effective work functions

**Fig 4:**
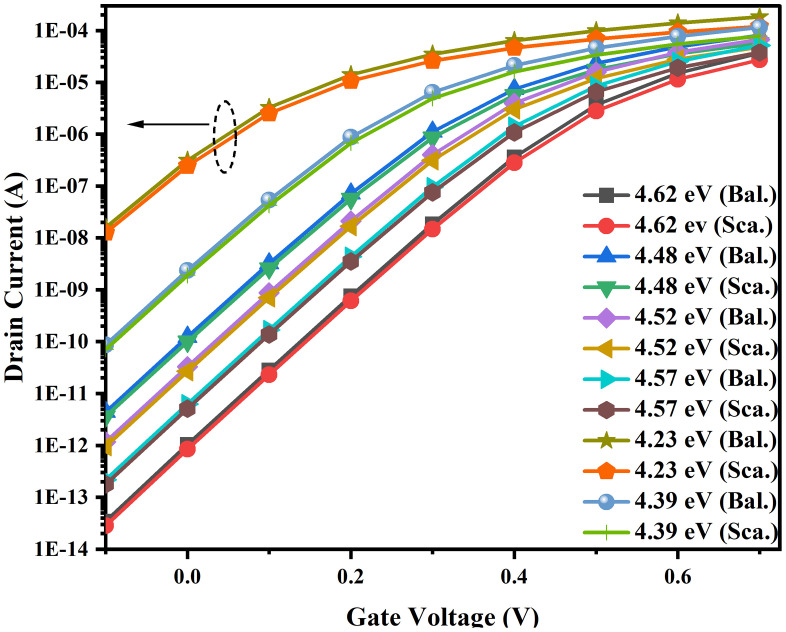
Comparison of the drain current and sub-threshold performance of the NSFET with varied EWF. The variation in the EWF is due to the presence of oxygen vacancies at one or more interfaces.

The numerical variation in the effective work function (EWF) of the gate stack region due to the presence of vacancies is discussed in our previous work [31]. This section primarily examines the impact of the vacancies on the scattering and sub-BTE transport mechanisms. Oxygen vacancies (OVs) can be modeled in TCAD as fixed oxide traps and interface traps in NSFET devices. In the bulk oxide region, a charge concentration of 1 × 10^18^ cm^−3^ is used to position the OV, while at the interfaces, the trap density is varied from 1 × 10^11^ cm^-2^ to 1 × 10^13^ cm^−2^ [31]. As with bulk impurities, neutral interface traps act as donors and become positively charged after donating an electron. Donor traps typically lie in the lower part of the bandgap, whereas acceptor traps become negatively charged after accepting an electron and exist in the upper region of the bandgap. When a voltage is applied to the gate, the Fermi level shifts up or down relative to the interface trap levels, leading to charge transfer within the interface traps. The presence of interface trap charges (ITCs) at interfaces increases the thermal density in the device and severely impacts material parameters, including the bandgap, intrinsic carrier concentration, density of states, and carrier mobility [38]. Acceptor traps in the device capture electrons at the interface traps and in the bulk traps, where the trapped electrons attract holes at the defined interfaces. In TCAD, simulations are performed based on the positions of trap levels within the bandgap and their concentrations. The Fig 4 summarizes the effects of various transport models on the transfer characteristics of the NSFET. The presence of vacancies is considered at the Si/SiO_2_/HfO_2_/TiN interfaces (corresponding to the EWF – 4.23 eV) and individual interfaces such as Si/SiO_2_, SiO_2_/HfO_2_, and HfO_2_/TiN. Vacancies at various interface sites introduce local lattice distortions and create a potential well or perturbation in the eMob. This affects the mean free path of the charge carriers, which, in turn, affects the eMob and thermal conductivity of the device [39–42]. Vacancies also introduce defect states in the bandgap (depending on the Fermi level position), acting as deep and surface interface traps with varying trap concentrations. From the ab-initio calculations, the EWF for the defect-free gate stack is computed to be 4.62 eV. The oxygen vacancies enhance the scattering in the device due to increased Coulomb (fixed charge), phonon (HfO_2_ being highly polar), and surface scattering. The vacancies create charged sites in the gate oxide due to displacement defects at the interface and in the channel. These charged sites interact electrostatically with the charge carriers, creating a localized electric field that reduces eMob. Additionally, oxygen vacancies contribute to lattice irregularity and surface roughness, thereby increasing the scattering time and the collision frequency [43,44]. Overall, this lowers eMob and degrades the drive current of the MOS device. When comparing the electrical performances in the two cases, i.e., with and without defect states, it is noted that V_T_ decreased by 2.5%, while the leakage current degraded by approximately five orders of magnitude. The I_D_ in diffusive transport is reduced by approximately 25% compared to the sub-BTE transport model, due to the combined effects of Coulombic, phonon, and surface roughness scattering at the defect-free gate stack (EWF – 4.62 eV). The scattering effect becomes more pronounced when we combine the impact of vacancies and scattering within the device, resulting in a degradation of the I_D_ by approximately 35%.

**Fig 5:**
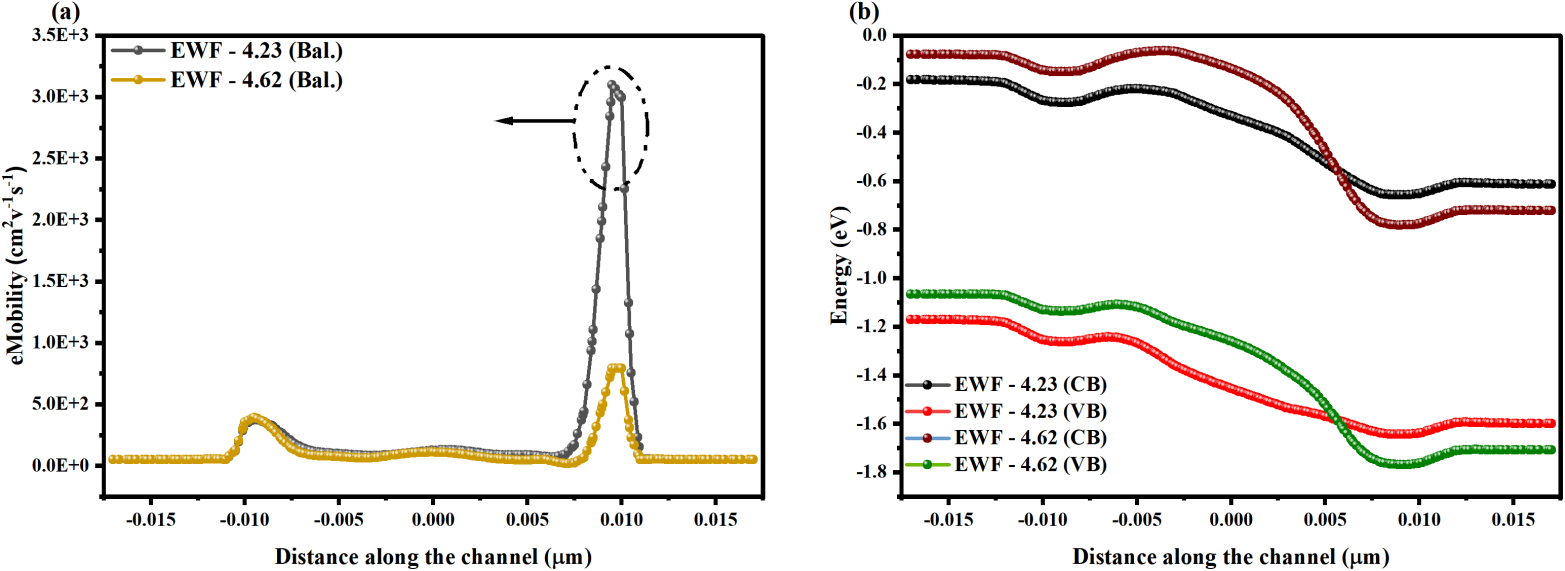
Profile showing (a) electron mobility along the channel in the NSFET, and (b) band-energy profile, showing the behavior of energy levels in defect and defect-free states.

**Fig 6:**
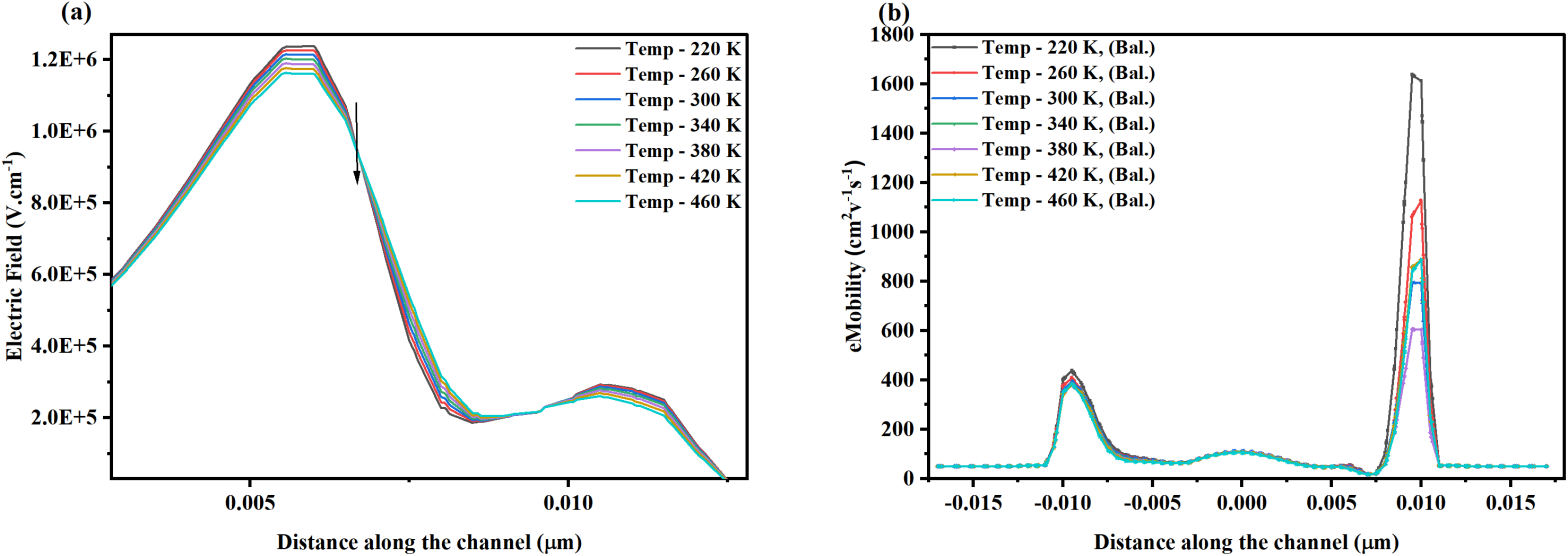
Impact of varied temperature from 220 K to 460 K on the (a) electric field, and (b) electron mobility, taken along the axis in sub-BTE transport mode. It can be noted that the electric field and mobility decrease at high temperatures due to electron-phonon interactions.

The scattering mechanisms are observed to be higher near the source-channel and channel-drain interface regions. This reduces the potential energy barriers and increases the probability of direct tunneling in the device, enhancing leakage current by three to five orders of magnitude, as presented in Fig 4. The defect states and traps formed at the oxide interface can also cause transverse tunneling due to the thin oxide layer and increased thermal carrier generation, ultimately leading to increased leakage current [45]. In contrast to typical scenarios, the eMob is higher (850 cm^2^v^-1^s^-1^) at the source-channel and channel-drain interfaces, as shown in Fig 6(a), due to increased electric fields and differences in potential gradients (Gaussian doping profile). The variance in the electric field accelerates charge carriers and enhances the eMob in the device. Typically, surface scattering leads to mobility degradation and increased leakage current, but, as shown in Fig 5(a), the eMob is improved at the interfaces by incorporating sub-BTE transport into the designed mobility framework. In the sub-BTE transport regime, the charge carrier tunnels with negligible scattering, and additionally, a high electric field at the interface also causes a strong injection velocity, which is largely maintained in the channel region, improving the eMob profile.

The enhanced leakage current in the case when all the interfaces of the gate stack have oxygen vacancies (EWF – 4.23 eV) can be attributed to the direct tunneling due to reduced conduction band offsets. In addition, there is trap-assisted tunneling (TAT), in which traps create a discrete energy level within the bandgap and reduce the energy difference between the trap level and the conduction band edge [45]. As presented in Fig 5(b), a steep band-bending profile near the channel-drain region enhances the carrier tunneling probability. The altered band profiles affect the terminal characteristics of the device, such as subthreshold swing and threshold voltage. The small valley near the band edge can affect the off-state current and may lead to charge-carrier scattering.

### 3.2. Impact of varying the ambient temperature on the electron mobility and lateral electric field

**Fig 7:**
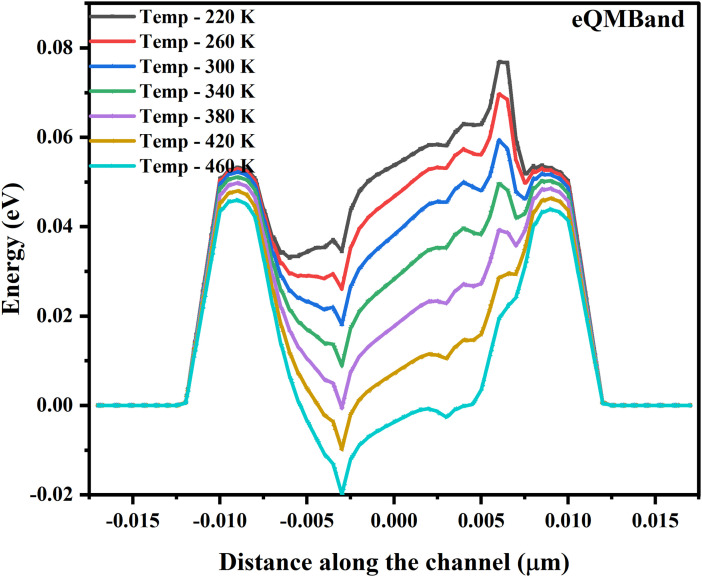
Profile depicting the variation in the band-gap energy due to the quantum mechanical effects at various temperatures in the NSFET.

The ambient temperature of the NSFET ranges from 220 K to 460 K to investigate the impact on the eMob in sub-BTE and diffusive transport mechanisms. Due to ultra-scaling in the nanodevice range, the total number of devices fabricated on a chip increases, leading to higher ambient temperatures and a more vertical power distribution. The high ambient temperature raises the lattice temperature, inducing variations in terminal characteristics, including eMob [46]. The changes in eMob affect I_D_, threshold voltage, and leakage currents. From the Fig 6, it can be observed that as the ambient temperature in the device increases, the magnitude of the electric field profiles in the channel region decreases. The electric field profiles in the channel region depend on doping concentrations and scattering events. Variation in the ambient temperature primarily triggers the phonon scattering in the device. Lattice vibrations increase with increasing temperature, leading to stronger interactions with charge carriers and thereby increasing the scattering rate and frequency. The peak electric field rate (1.2 × 10^6^ V.cm^-1^) is observed at 220 K at the drain-channel interface region due to the asymmetric potential distribution and strong inversion layer. The lower source potential creates a steeper potential gradient near the source-channel region, resulting in a strong electric field in the region. When the ambient temperature increases to 460 K, the magnitude of the electric field decreases by about 8%. When the NSFET operates at extremely low temperatures (220 K), the lateral electric field profile sharpens at the drain/channel interface due to the clustering of scattering events in the region, giving it a quantum well shape as depicted in Fig 7. The lateral electric field transports charge carriers from source to drain. In contrast, the vertical electric field induced by the supply voltage is responsible for forming the inversion layer in the device. Usually, at low ambient temperature, the increased threshold voltage reduces the effective gate overdrive voltage and weakens the vertical electric field [47,48].

In contrast, the lateral electric field varies along with the channel in the saturation region due to the pinch-off and is amplified by the higher eMob, leading to a stronger electric field near the drain. The eMob profile along the channel region, as shown in Fig 6(b), indicates that, in the sub-BTE transport regime, carrier mobility decreases with increasing temperature. The primary reason behind this slump is the dominant phonon scattering. The lattice vibration increases at high temperature (450 K). Due to this, the scattering time and frequency increase, and the electron-phonon interactions are enhanced. Overall, it increases phonon scattering, which reduces the eMob. High-temperature phonon scattering reduces the mean free path and adversely affects the effective injection velocity [49]. Surface roughness and Coulomb scattering also impact mobility by elevating the thermal energy and tunneling of electrons via the thin interfacial oxide layer. The peak eMob (1641 cm^2^v^-1^s^-1^) in sub-BTE is noted to be at 220 K, and 450 K, it drops to 900 cm^2^v^-1^s^-1^, causing a decrement of ~ 45% in the magnitude. Overall, the performance deviation in the eMob is calculated to be at 328.7 cm^2^v^-1^s^-1^; these standard deviations in performance, together with reduced eMob, lower the I_D_ and impact the reliability of the NSFET. The bandgap shift due to quantum-mechanical effects is shown in Fig 7. This bandgap shift profile shows a reduction in the bandgap energy, mainly due to electron-phonon interactions at higher temperatures. The eQM band shift is modeled using the Schrödinger-Poisson solver and the density-gradient model in Sentaurus TCAD.

The plot shows that the biasing supply lowers the potential profile in the channel region, creating a quantum well for the electrons, and the source/drain region forms the potential energy barriers. As the temperature increases from 220 K to 460 K, the energy band profile and the barrier height near the source/drain region decrease. The minimum energy barrier is near the 0 eV, and the peak is reached at 0.07 eV. Usually, the bandgap decreases with increased operational temperature due to the electron-phonon interactions. The temperature range is 220–460 K, corresponding to a band-gap reduction of 48–72 meV. The downward shift of the band profiles suggests strong bandgap narrowing modified by geometrical confinements. When the NSFET operates at low temperatures (e.g., 220 K, 260 K), geometric confinement effects are more pronounced due to lower thermal energy, and charge carriers are more likely to occupy quantized energy levels. Thermal energy increases when the temperature is high (460 K), and charge carriers occupy high-energy states, which causes the band gap narrowing [50–52].

**Fig 8:**
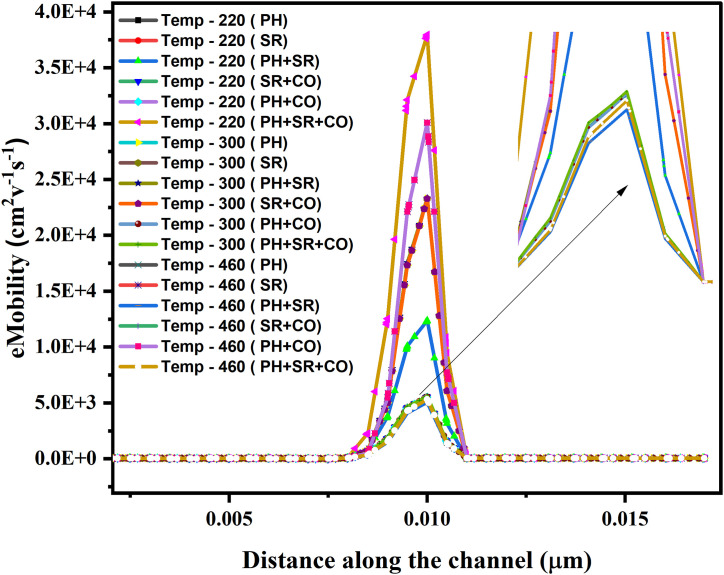
Impact of various scattering (individual and combined effects) on the electron mobility with variation in the temperature.

The simulated electron mobility profiles along the NSFET channel exhibit two peaks in electron mobility, localized at the source-channel and channel-drain junctions, at all the temperatures (220 K, 300 K, 460 K) and across various scattering configurations as shown in Fig 8. Initially, the presence of various dominant impurity scattering in the highly doped source and drain regions reduces electron mobility in these regions [53]. In the channel region, mobility increases at the source-channel and channel-drain interfaces, reflecting the abrupt reduction in ionized impurity scattering across the doping gradient [54]. It can be noted that the combined effects of the phonon, surface roughness, and coulombic (PH + SR + CO) scattering mechanism yield the lowest peak mobility at 300 K and 460 K, supporting the claims from Matthiessen’s rule [55]. In contrast, the PH + SR + CO scattering at 220 K observes a higher electron mobility than at 300 K and 460 K. The notable increase in mobility at 220K can be due to the reduced phonon scattering at low temperatures. In a low-temperature operating region and at reduced channel length, charge-carrier transport is governed by injection velocity and energy-relaxation length rather than the traditional diffusive transport mechanism and momentum relaxation events [48,55]. It can be inferred from the observation that at 220 K, the electron mobility profile doesn’t reflect intrinsic scattering-limited mobility, but rather the dominance of quasi-ballistic transport and non-local carrier dynamics. The individual effects of PH, SR, and CO scattering on the observed mobility are small and nearly identical, due to the low electric-field profiles at the junction interfaces, and the charge carriers remain near quasi-equilibrium. It can also be observed that the variation in the electron mobility profiles for combined scattering mechanisms (PH + SR, PH + CO, and SR + CO) is due to the distinct physical nature and spatial influence of the underlying scattering mechanisms. In the PH + SR scattering, phonon scattering governs carrier energy relaxation, while surface roughness scattering introduces strong short-range momentum degradation at the semiconductor–oxide interface, leading to a suppressed mobility peak near the channel–drain junction due to reduced carrier acceleration under high confinement, as shown in Fig 8 [54–56]. In the case of PH + CO scattering, charge impurities and interface traps induce Coulomb scattering, leading to fluctuations in the electrostatic potential profile in the device and disrupting charge-carrier transport in the channel. The effects of PH + CO scattering result in a spatially uniform reduction in mobility and a much-reduced and asymmetric peak profile at the channel-drain interface. The strongest mobility degradation occurs in the combined SR + CO scattering state, where both localized interfacial scattering and spatially extended Coulomb interactions from charged defects are present [57,58]. This results in the degraded low-field mobility and a reduced peak near the channel–drain junction.

### 3.3. Impact of various gate lengths

**Fig 9:**
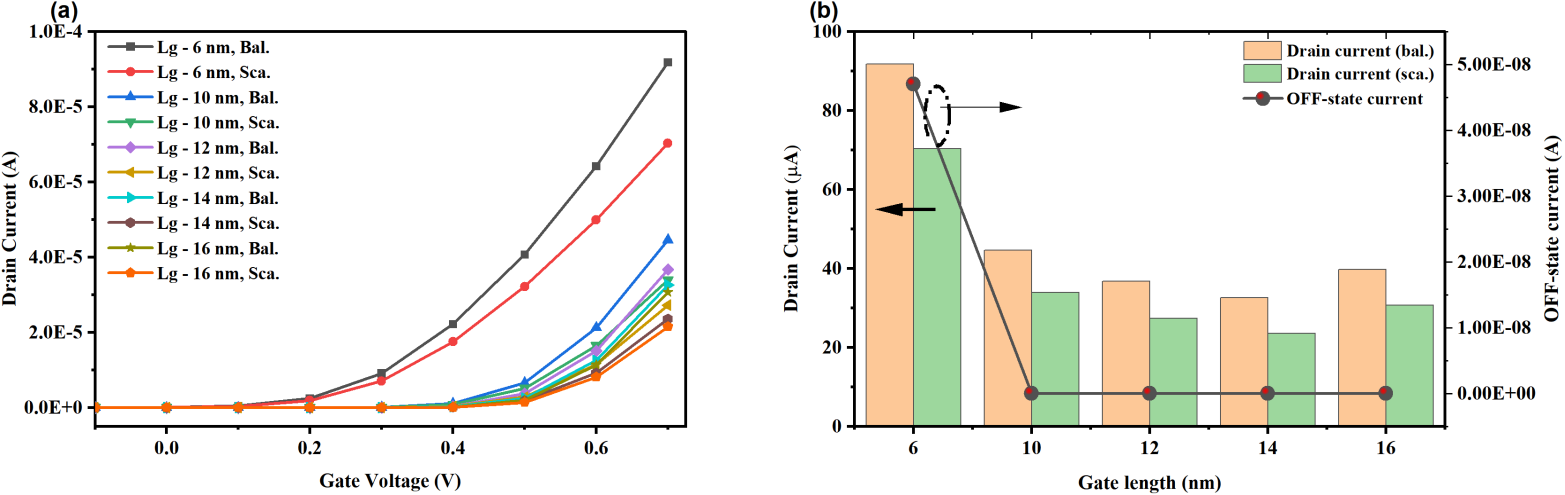
(a) transfer characteristics with the gate length scaling in sub-BTE and diffusive transport mode, (b) variation in the drain current and leakage current due to the scattering in the device.

This section aims to study scattering and the sub-BTE phenomenon in a 6-nm-gate-length NSFET device. The study incorporates the sub-BTE transport model along with the diffusive transport model. To achieve a comprehensive comparison and understanding, we varied the gate length from 6 to 16 nm, using the 12 nm-based device as the benchmark [59]. The computed transfer characteristics are shown in Fig 9(a), and the extracted results are tabulated in Table 2 and also depicted in Fig 9(b). It can be noted that the scaling of gate length causes a reduction in V_T_ due to the threshold voltage roll-off and enhances leakage current.

**Table 2:** Tabulated results for the gate length scaling of the scaled NSFET device.

Gate length	mode	V_T_ (V)	SS (mV/dec)	I_D_ (A)	I_OFF_(A)
12	Sca	0.447	68.7	27.3 × 10^−6^	8.53 × 10^-13^
	Bal	0.436	68.6	36.7 × 10^−6^	1.04 × 10^-12^
14	Sca	0.464	65.3	23.5 × 10^−6^	1.62 × 10^-13^
	Bal	0.454	65.2	32.6 × 10^−6^	2.01 × 10^-13^
16	Sca	0.473	63.4	21.5 × 10^−6^	5.04 × 10^-14^
	Bal	0.463	63.3	30.7 × 10^−6^	6.38 × 10^-14^
10	Sca	0.411	73.8	33.9 × 10^−6^	9.18 × 10^-12^
	Bal	0.398	73.7	44.6 × 10^−6^	1.11 × 10^-11^
6	Sca	0.194	104.6	70.3 × 10^−6^	3.75 × 10^−8^
	Bal	0.178	103.5	91.8 × 10^−6^	4.76 × 10^−8^

The drive current increases with downscaling of the gate length, due to reduced scattering and a shorter source-to-drain distance in the device. A similar enhancement pattern in the I_D_ is also observed in the sub-BTE transport model, due to the reduced source-drain distance. Leakage current increases in the device with a downscaled gate length, representing a performance trade-off. The primary reasons for the increase in leakage current are threshold-voltage roll-off and reduced gate control over the channel region. The reduction in I_D_ during diffusive transport is 23% at the 6 nm gate length. The results in Table 2 compare transistor performance across gate lengths (6–16 nm) in sub-BTE (Bal.) and scattering (Sca.) transport modes, focusing on key terminal characteristics of the NSFET. At 12 nm gate length, the sub-BTE transport performs better than scattering models (diffusive transport mode), demonstrating a 2.46% lower V_T_, and 34.43% higher I_D_, but with a 21.92% higher. As the gate length decreases to 6 nm, both scattering and sub-BTE transport modes exhibit an increase in the V_T_, SS, I_D_, and I_OFF_. The leakage current increases by five orders of magnitude and degrades the I_ON_/I_OFF_ ratio. This indicates challenges in ultra-scaled transistors due to enhanced scattering and tunneling effects [60,61]. Traditionally, sub-BTE transport enables unimpeded carrier flow, reducing V_T_ and enhancing I_D_, but the increased I_OFF_ at shorter gate lengths suggests tunneling through thinner barriers [62]. Scattering mode, influenced by phonons, surface roughness, and Coulombic interactions, hinders carriers, lowering I_D_ and slightly increasing V_T_ and SS. Higher electric fields and proximity to the interface amplify scattering at shorter gate lengths, significantly worsening SS and I_OFF_, especially at 6 nm. These trends highlight the trade-offs in nanoscale transistors, where sub-BTE transport offers performance advantages but struggles with leakage, while the scattering mechanisms limit drive current and subthreshold control as scaling continues [61,63,64].

**Fig 10:**
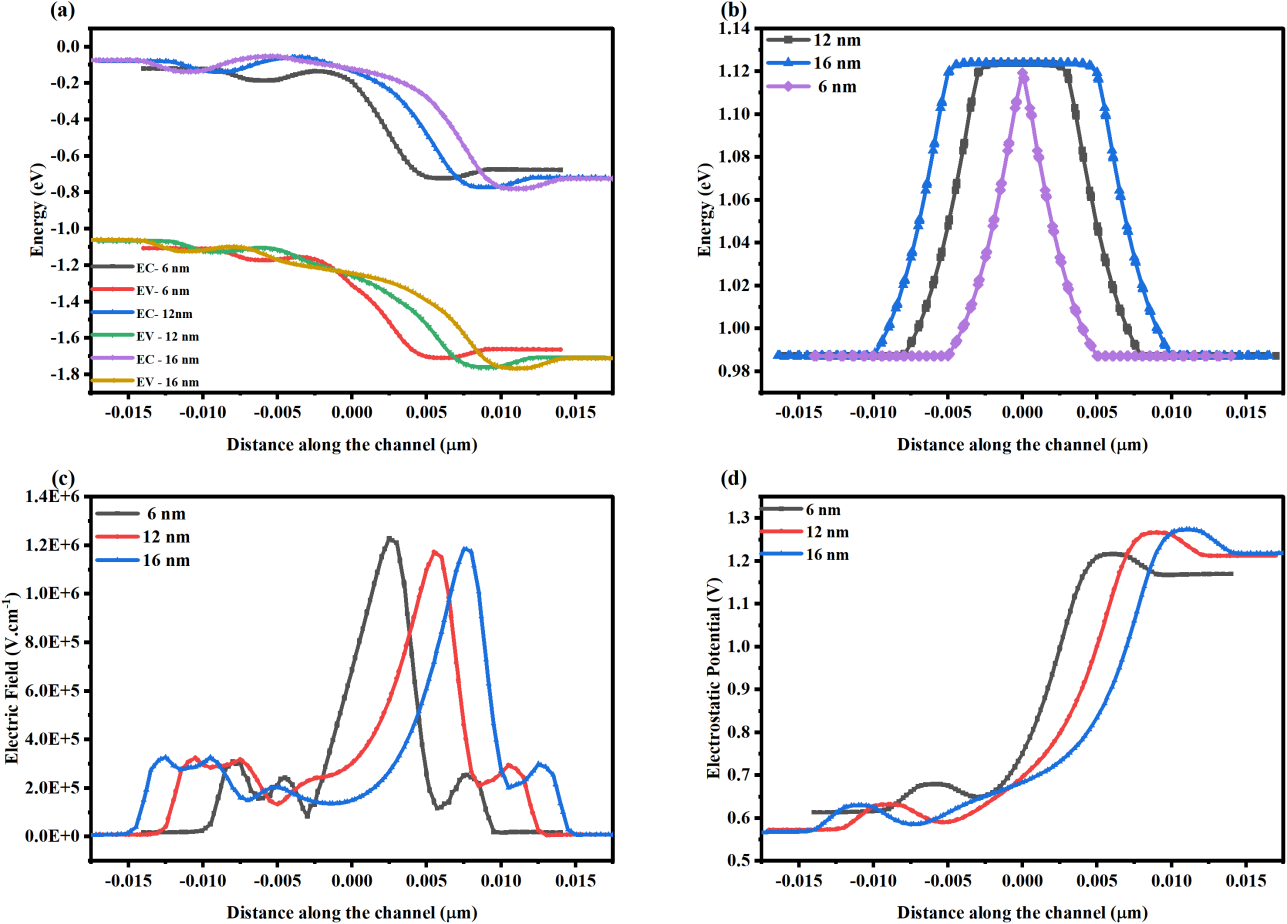
Variation in the terminal behavior of the NSFET due to the gate length scaling on (a) energy band profile, (b) bandgap narrowing, (c) lateral electric field, (d) electrostatic potential.

Fig 10(a-d) shows the various electrical performance metrics to understand the scaling phenomenon across gate lengths from 16 to 6 nm. Fig 10(a) shows that the band bending is sharper at the 6 nm gate length than at the 12 and 16 nm gate lengths in the NSFET. Band bending in the conduction and valence bands near the channel-drain region increases the probability of electron tunneling, thereby increasing leakage current. The reduction in the energy barrier is due to the high doping concentration and a reduced CPP in the device. The peak lateral electric field (1.2 × 10^6^ V. cm^-1^) in the channel region of the 6 nm gate length based NSFET leads to higher quantum confinement. In this case, the charge carriers are confined to a narrow potential well, leading to a thinner depletion region at the channel-drain and drain-substrate interfaces. This alters the band alignment at the source-channel interface at high drain bias, leading to a reduced I_ON_/I_OFF_ ratio.

The combined impact of scattering (phonon + surface roughness + coulombic scattering) is more profound at a smaller gate length (6 nm) than at a higher gate length (16 nm) due to reduced contact poly pitch (CPP). The impact of the individual scattering mechanisms is shown in Table 3.

**Table 3:** Comparative analysis of the figure of merit (electrical characteristics at the terminal nodes) for 12 nm and 6 nm gate-length stacked NSFETs under various transport mechanisms. Note Bal. represents the sub-BTE transport mechanism, Ph is phonon scattering, CO is Coulombic scattering, and SR is for surface roughness scattering.

Gate length	mode	V_T_ (V)	SS (mV/dec)	I_D_ (A)	I_OFF_ (A)	I_ON_/I_OFF_ (A)
12	Bal.	0.436	68.6	36.7 × 10^−6^	1.04 × 10^-12^	10^7^
	PH + SR + CO	0.447	68.7	27.3 × 10^−6^	8.53 × 10^-13^	10^7^
	PH + SR	0.444	68.6	29.1 × 10^−6^	8.70 × 10^-13^	10^7^
	PH	0.444	68.6	30.1 × 10^−6^	8.80 × 10^-13^	10^7^
6	Bal.	0.178	103.5	91.7 × 10^−6^	4.76 × 10^−8^	10^3^
	PH + SR + CO	0.194	104.6	70.3 × 10^−6^	3.75 × 10^−8^	10^3^
	PH + SR	0.186	103.5	77.7 × 10^−6^	4.19 × 10^−8^	10^3^
	PH	0.185	103.5	79.7 × 10^−6^	4.22 × 10^−8^	10^3^

It is observed that sub-BTE transport exhibits minimal scattering, enabling carriers to traverse the channel without energy loss, resulting in the highest I_D_ of 36.7 μA at 12 nm and 91.7 μA at 6 nm. In contrast, phonon scattering (PH) introduces energy loss through carrier interactions with lattice vibrations, reducing I_D_ (e.g., 30.1 μA at 12 nm, 79.7 μA at 6 nm) compared to sub-BTE transport. The inclusion of surface roughness scattering (PH + SR) and Coulombic scattering (PH + SR + CO) further degrades transistor performance due to surface irregularities at the channel interface and varying trap levels, ultimately reducing carrier mobility. I_OFF_ is marginally reduced, possibly due to increased effective resistance from surface roughness and coulombic interactions. The combined effect of all the scattering mechanisms (PH + SR + CO) included in the study yields the lowest I_D_, due to local electric-field scattering of carriers and charged impurities.

## 4. Conclusions

In summary, we investigated sub-BTE and diffusive transport mechanisms, focusing on the individual impacts of prominent scattering models (PH, SR, and CO) on the electrical performance of the NSFET. We also explored the behavior of the NSFET at scaled channel lengths and varying temperatures within the diffusive and sub-BTE transport framework. The design and simulation of the transport mechanisms are conducted using Sentaurus TCAD models, and the device characteristics are calibrated against an experimental device to ensure reliability. The ab initio results show that interface and bulk vacancies in the Si/SiO_2_/HfO_2_/TiN gate stack lead to a variation of approximately 400 meV in the effective work function. The terminal behavior of the device degrades due to V_T_ roll-off, and an enhanced carrier tunneling probability results in high leakage current. A high lateral electric field and potential gradient increase electron mobility (850 cm²/V·s) at the drain-channel interface, where it is typically reduced by surface roughness scattering. Increasing the ambient temperature of the NSFET enhances the lattice temperature and can increase the phonon scattering rate. The enhanced lattice vibrations lead to strong electron-electron and electron-phonon interactions at high temperatures, reducing electron mobility and increasing leakage current. The clustering of electrons in the drain-channel region causes a sharp increase in the absolute electric field of the NSFET at 220 K, driven by strong inversion and an asymmetric potential distribution of electric fields in the device. The increased scattering rate at high temperatures distorts the bandgap profiles, varying it from 48 to 72 meV. When the gate length is scaled from 16 nm to 6 nm, reduced V_T_ and increased leakage current are observed due to geometric confinement, which enhances the band-to-band tunneling probability. The study also maps the impact of individual scattering events on the scaled 6 nm gate-length NSFET device, finding that the sub-BTE mechanism exhibits minimal scattering. Electrical behavior is influenced by phonons, surface roughness, and Coulombic interactions, which hinder the charge carrier mobility. High lateral electric fields and interface proximity elevate the scattering rate at shorter gate lengths, significantly worsening SS and I_OFF_. The combined effects of all the scattering models result in the lowest drain current and pose a reliability challenge for further device scaling. Thus, a reduced gate length of 6 nm may be detrimental to device performance and reliability.

## Supporting information

S1 Data Supplementary Data. (ZIP)
